# Plasma cells in and around the central nervous system

**DOI:** 10.3389/fimmu.2025.1735430

**Published:** 2026-01-08

**Authors:** Alexander K. Merder, E. Ashley Moseman

**Affiliations:** Department of Integrative Immunobiology, Duke University, Durham, NC, United States

**Keywords:** CNS humoral immunity, CNS antibody, antibody secreting cell (ASC), plasma cell (PC), CNS infection, CNS autoimmunity

## Abstract

Secreting a continuous, and sometimes life-long antibody supply, plasma cells are the effector arm of humoral immune system. With an incredibly diverse array of binding specificities, antibodies play critical roles in homeostasis and disease. Traditional views of plasma cells have them function at a distance, relying on circulation to ferry their antibodies to peripheral tissues. However, this review focuses on plasma cells that operate locally within tissues that lack ready access to circulating antibody, specifically, we explore plasma cells that accumulate within the central nervous system (CNS) and its borders. Through both antibody secretion and immunoregulation, plasma cells impact responses to neuroinvasive pathogens, CNS-targeting autoimmune diseases, and CNS tumors. In border sites, like the meninges and olfactory mucosa, plasma cells serve to protect against CNS pathogen invasion while also mediating pathology in autoimmunity and inflammatory diseases. In considering plasma cells in and around the CNS, we discuss their localization, function, migration, local differentiation, and persistence. Importantly, we examine where gaps remain in our knowledge of CNS plasma cells and how this work will impact the prevention and treatment of CNS infection and autoimmunity.

## 1 Introduction

As the effector arm of the B cell lineage, plasma cells establish humoral immunity through antibody production and secretion. These antibodies are critical protective mediators against infection and yet play foundational roles in the pathogenesis of harmful inflammatory and autoimmune diseases (1, 2). Apart from their role in antibody production, plasma cells also secrete regulatory cytokines, like IL-10, indicating an ability to modulate immune responses beyond antibody production (3–5). Along with their roles during infection and autoimmunity, plasma cells are also important mediators in cancer responses, with well-documented protective anti-tumor and pathogenic pro-tumor regulatory functions (6–9). By harnessing plasma cells and their antibodies, millions of lives have been saved through vaccinations that protect against viral and bacterial disease (10–12). At the same time, therapeutics specifically targeting and depleting B cells and plasma cells are efficacious in treating several autoimmune diseases (13–15). In cancer, B cell-based immunotherapy to utilize anti-tumor humoral responses are gathering early clinical interest. While still under development, these could one day be used alongside long-standing immunotherapeutic treatments focused on eliciting effector T cell responses (16–18).

Plasma cell involvement in these different immune responses depends on their generation, a well-studied process that starts with activation of naïve and memory B cells by cognate antigen after which B cells typically interact with activated CD4 T cells to receive “help”. B cell activation is followed by proliferation and differentiation into a variety of cell fates, including germinal center (GC) B cells, memory B cells, and plasma cells (19–21). GC B cells, supported by T follicular helper cells, undergo extensive and prolonged proliferation, along with an iterative process of somatic hypermutation and BCR affinity maturation that generates increasingly high affinity plasma cells and memory B cells (20). Importantly, early after activation, B cells can undergo antibody isotype class switching that converts their immunoglobulin (Ig) isotype expression from IgM to IgG, IgA, or IgE to diversify antibody effector functions (22).

One area of increasing interest in plasma cell biology is their presence and function in tissues across the body. Apart from well-studied niches like the bone marrow and spleen, plasma cells migrate to mucosal barriers throughout the body, including the small and large intestine, the lung, the upper respiratory tract (URT), and the genitourinary tract (23, 24). Non-mucosal tissues like the meninges, visceral adipose tissue, and thymus have also been found to host plasma cells (24–27). In addition, plasma cells are often identified in actively infected and inflamed tissues where they provide directed local antibody secretion (28–30).

**Figure 1:**
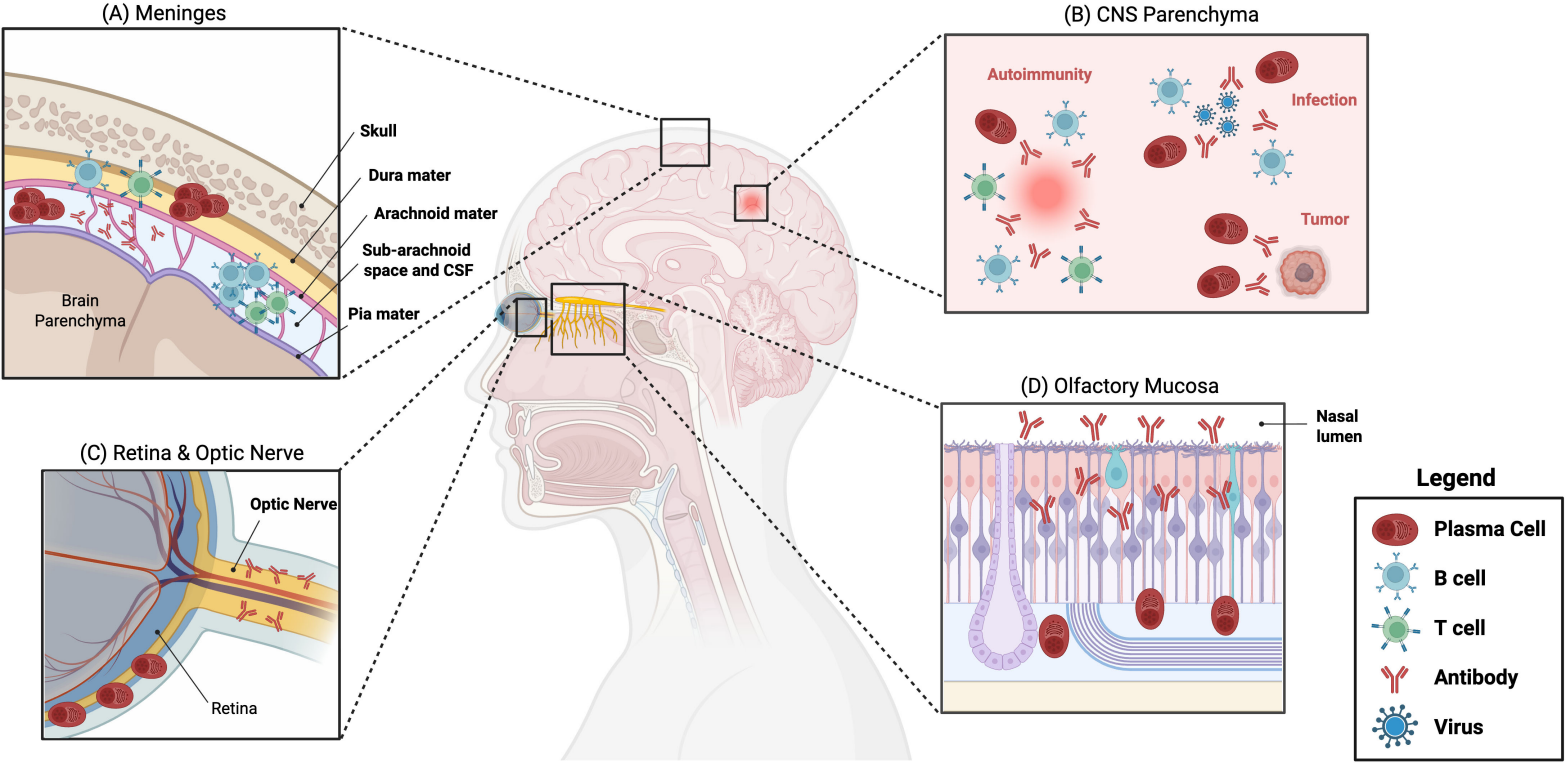
Plasma cells are present across CNS border tissues and within the parenchyma. **(A)** Meninges: In homeostasis, plasma cells are present in the dura mater or along the veinous sinuses, with IgA+ plasma cells dominating in young mice and IgG+ and IgM+ plasma cells increasing in frequency with age. B cells and T cells also localize in the tissue during homeostasis, with a notable accumulation of T-bet+ memory B cells in older mice. The leptomeninges and CSF are largely devoid of plasma cells in homeostasis; however, in infection and autoimmune inflammation, plasma cells and antibody accumulate in these regions. **(B)** CNS parenchyma: Similar to the leptomeninges, plasma cells are rarely found in the steady-state parenchyma; yet, during autoimmunity, infection, and CNS tumors, plasma cells accumulate at these sites within the parenchyma. Memory B cells and T cells are also often found at autoimmune lesions and at sites of infections. During both CNS autoimmunity and infection, antibody has been identified deposited on self-antigen (autoimmunity) and virally infected cells (infection). In tumors, IgG+ plasma cells can be pro-tumorigenic, as IgG-FcgRII-mTOR signaling induces proliferation in glioblastoma stem cells. **(C)** Retina & Optic Nerve: Both the retina and the optic nerve are important to consider in the context of CNS plasma cell involvement. In autoimmune uveitis and optic neuritis, the significance of plasma cells and their secreted antibody have been established yet remain understudied. In the retina, plasma cells have been identified in autoimmune inflammation and seen to play both regulatory and pro-inflammatory roles, with the direct role of antibody not yet determined. In the optic nerve, the pathogenesis of autoimmune-driven optic neuritis is known to involve antibodies, with some evidence in MS-driven disease suggesting plasma cells are present locally but little known about plasma cell presence during NMO-driven pathology. **(D)** Olfactory mucosa: In the olfactory mucosa, plasma cells have been identified in the tissue and are known to secrete antibody into the lumen to protect against viral infection. This antibody-based protection is crucial for preventing infection of the URT and spread from host to host as well as preventing subsequent neuroinvasion.

This review focuses on plasma cells that migrate to, or otherwise reside in and around, the central nervous system (CNS) during both homeostasis and inflammation (**Figure 1**). Substantial recent work has been done on the role of B lineage cells in CNS immunity especially along meningeal borders but also within the brain parenchyma. However, beyond the tri-layered meningeal immune compartment surrounding the CNS, several other neurological tissues are home to B cells and plasma cells (31). The retina and optic nerve are often overlooked CNS connections where plasma cells have been identified. Our group has a particular interest in the olfactory surface, where the olfactory mucosa (OM) forms a unique CNS barrier tissue within the upper airway (32). Clarifying how plasma cells localize, function, migrate, and persist within the CNS and its barrier tissues deepens our understanding of how they function in protective and pathological immune responses.

## 2 Plasma cells at CNS borders

To start, we will consider what is known about plasma cells in two tissues that border the CNS parenchyma; the meninges and olfactory mucosa. While these two tissues are quite unlike each other, they are both important CNS barriers, with humoral immunity being a critical part of their defenses. We will discuss what is known about plasma cells in these tissues during both homeostasis and inflammation.

### 2.1 Meninges and cerebrospinal fluid

In homeostasis, many B lineage cells exist in the outermost meningeal layer, the dura mater, while the more CNS proximal leptomeninges (arachnoid mater and pia mater) and cerebrospinal fluid (CSF) are largely devoid of immune cells (33–37). Plasma cells make up 0.3% of the immune cell population in the dura mater of young mice; however, aging leads to increased plasma cell frequency (33). Aged mice also acquire alterations in plasma cell isotype expression, with IgA+ plasma cells dominating in younger mice and IgG+ and IgM+ plasma cells accumulating with age (25, 33). T-bet expressing memory B cells with high levels of somatic hypermutation, and the potential for rapid differentiation into plasma cells, also accumulate in the meninges in aged mice (33, 38, 39). Importantly, IgA+ plasma cells were also identified in human dural tissue (25). Comparing how murine meningeal B cell and plasma cell populations relate to humans, given differences in environment and pathogen exposure, will be an important focus of future research.

Interestingly, the IgA+ plasma cells present in the dura mater appear to originate from the gut and are not present in germ free mice, indicating microbiota are involved in their generation (25). These plasma cells are positioned along the dural venous sinuses, where fenestrated capillaries and relatively slow blood flow combine to increase the risk for blood-borne pathogen CNS access (25, 36). Plasma cell localization to an area of vulnerability, alongside experiments specifically depleting meningeal plasma cells, suggested an important role for these cells in protecting the CNS against hematogenous pathogens. In addition, recent work has identified lymphocyte clusters in the dura mater, termed dural associated lymphoid tissue (DALT), that exist during homeostasis and expand with age and local or systemic challenge (40). These lymphoid hubs were found to contain plasma cells that likely arise locally (40). Although the relative importance of DALT plasma cells compared with those generated in tissue draining secondary lymphoid organs remains unclear, plasma cells generated locally may be important for CNS defense in a similar manner to those residing along the dural veinous sinuses. In this way, plasma cells that differentiate in the DALT could impact CNS invasion by systemic pathogens.

During CNS inflammation due to either infection or autoimmunity, plasma cells accumulate in the meninges and CSF, resulting in vastly increased intrathecal antibody. The earliest data suggesting plasma cell accumulation in these sites was the discovery of oligoclonal bands, discrete bands of immunoglobulins enriched in the CSF over peripheral serum (41–46). Clonally expanded plasma cells have since been directly identified in the CSF and shown to produce the antibodies detected in oligoclonal bands (47–55). Importantly, antibodies produced and found in the CSF are specific for relevant self and viral antigens depending on the inflammatory context (44, 54, 56–61). In fact, antibodies isolated from Multiple Sclerosis (MS) patient CSF samples can induce demyelination on ex vivo spinal cord explants, suggesting a direct functional relevance in disease pathology (62). Oligoclonal bands, and clonally expanded plasma cells, in the CSF also suggest that antigen-specific B cells enter the CNS and are likely triggered to differentiate into plasma cells by local antigen engagement. This cognate antigen-driven plasma cell generation locally in the CNS tissue would explain why there is discrete enrichment of either self or virus-specific antibodies as opposed to uncontrolled migration of “nonspecific” plasma cells from circulation. Whether plasma cells are present in the CSF as a consequence of general CNS accumulation or if they actively migrate or locally differentiate is a question that requires further study.

B cell-rich aggregates that contain plasma cells commonly form in the leptomeninges of patients with CNS-targeting autoimmune diseases (63–69). These cellular clusters have been identified in MS patients and mouse models of demyelinating autoimmune disease (70). CNS immune aggregates show varying organization levels, from simple, disorganized B cell groupings to organized microanatomical structures resembling zonally defined lymphoid follicles (63, 64, 67). While increased meningeal inflammation and the presence of B cell aggregates is often associated with more severe pathology and disease, the exact role for plasma cells in these CNS border sites is not fully understood (64, 68). Meningeal B cell accumulation is further associated with adjacent subpial cortical demyelination implicating these B lineage cells on the CNS borders in parenchymal pathology (66, 71). In addition, anti-CD20 B cell depletion therapy, which shows efficacy in treating MS, has been shown to disrupt meningeal B cell aggregates and reduce other CNS-infiltrating lymphocytes (15, 72). Thus, disrupting these meningeal B cell aggregates that house local plasma cells may play a role in determining the efficacy of B cell depletion strategies.

Despite these associations with pathology and efficacy, how plasma cells within the leptomeninges and/or CSF mechanistically impact parenchymal pathology is still unclear. Interestingly, B lineage cells found in the meninges and CSF are clonally related to those found in MS parenchymal lesions, suggesting a functional overlap between border and parenchymal populations (73, 74). Are cells in the meninges causing disease via antibody production into CSF or do meningeal plasma cells then migrate into the parenchyma to cause disease? Or does the meninges support both cellular and antibody movement into the CNS? Functionally distinguishing these possibilities will require future experimentation to directly test them. Unraveling how plasma cell accumulation (via migration or local generation) within the meninges and CSF impacts disease pathology will be crucial to understanding their protective and pathogenic functions.

### 2.2 Olfactory mucosa

The olfactory mucosa lies in the posterior nasal mucosa within the upper airway, directly adjacent to the CNS. To detect odorant molecules, olfactory sensory neurons residing in the airway, project their axons through the cribriform plate at the front of the skull into the olfactory bulb of the brain parenchyma (32). This remarkable anatomy empowers rapid chemosensory recognition of odors but also undermines fundamental CNS barriers like the blood brain barrier and skull. Both intra- and extracellular pathogens can hijack the olfactory route to invade the CNS tissue (75–79). Plasma cells in the olfactory mucosa are understudied both for their role in mucosal barrier immunity and as players in CNS protection from neuroinvasive pathogens. While many groups have studied URT humoral immunity, very few have specifically focused on the olfactory mucosa as a distinct tissue, separate from the respiratory regions (32).

Besides the fact that the olfactory mucosa is uniquely adjacent to the brain parenchyma and highly neuronal, the two zonally distinct URT regions are functionally differentiated by the ability of circulating antibodies to access and protect the mucosal tissue. Recent work from our group demonstrated that while circulating antiviral antibodies can reach and protect the respiratory tissue, the vascular endothelium within the olfactory mucosa creates a blood olfactory barrier (BOB) that excludes potentially protective serum antibodies (80). Other work has shown variability in the protective capacity of passively transferred antibodies in URT influenza infections which is consistent with protection in only respiratory portions of the nasal mucosa (81, 82). Importantly, despite exclusion of circulating antibodies, plasma cells can migrate into the olfactory tissue for local, extravascular antibody production that effectively protects against viral mucosal infection and subsequent brain invasion (80). Antiviral protection by olfactory plasma cells is dependent upon activation-induced cytidine deaminase (AID) expression, CD4 T cell help, and CXCR3 signaling; however, many aspects of olfactory plasma cell biology remain unexplored, such as their longevity and what signals control their migration and residence (80).

Furthermore, single cell sequencing studies have identified plasma cells, among other B lineage cells, specifically in the olfactory mucosa (83–85). After nasal vaccination, IgA-expressing B lineage cells originating from local lymphoid tissue can be programmed to migrate into glandular tissues of the upper airway (86). This process is driven by CCL28 expression (86), an observation consistent with lung studies demonstrating a glandular plasma cell niche formed by CCL28 (87). It is unclear whether the CCL28-CCR10 axis is important for localization within non-glandular URT areas, and specifically within olfactory tissues. However, in a study of human olfactory tissue, B lineage cells expressing *Jchain* as well as IgA and IgM were found adjacent to secretory Bowman’s glands, localization that is potentially important for delivering antibody into the nasal lumen (88).

A substantial number of studies have identified plasma cells and mucosal antibody (particularly IgA) within human and murine nasal swabs, washes, and biopsies, after infection and vaccination; however, the technical collection methods used prevent conclusions on whether the cells and antibodies were derived from olfactory tissue (89–95). Recent work also found memory B cells in human nasal swabs, suggesting a source of cells with the potential to locally differentiate into URT plasma cells (96). Similarly, in mice, ectopic GC-like aggregates were recently identified in the nasal mucosa following influenza infection, yet their consequences during olfactory humoral immunity remain to be determined (97). In chronic rhinosinusitis, a chronic URT inflammatory condition, plasma cells and B cells are present and implicated in the disease course (98–101). It is important to note that chronic rhinosinusitis can cause smell loss, but there is no direct evidence that plasma cells or B cells associated with disease are within the olfactory tissue.

Overall, more work is needed to better understand olfactory plasma cell biology as well as their translatability to human health and disease. Their localization at the CNS mucosal barrier suggests olfactory plasma cells likely play important roles in protecting against a variety of CNS pathogens. While SARS-CoV-2 was not directly neuroinvasive, the ability to replicate within olfactory tissues placed this pandemic virus dangerously near the CNS (102, 103). Vaccinations saved countless lives during the SARS-CoV-2 pandemic, yet these vaccines provided relatively poor upper airway protection, indicating that we need to enhance our understanding of basic plasma cell biology within the olfactory mucosa if we are to improve vaccinations for a future neuroinvasive pandemic.

## 3 Plasma cells within the CNS parenchyma

Like the leptomeninges and CSF, B lineage cells, including plasma cells, are largely excluded from the CNS parenchyma in homeostasis but dramatically increase in frequency during infection and autoimmunity. In many CNS viral infection models, including West Nile virus, Sindbis virus, JHMV, LCMV, and murine cytomegalovirus, both B cells and plasma cells can be identified within the CNS parenchyma (47, 104–113). These B lineage cells are largely virus-specific and express several different Ig isotypes, as the CNS immune response parallels that expected for peripheral infections, with IgM dominating the early antibody responses before making way for IgG and IgA (106, 107, 112). Interestingly, plasma cells responding to Sindbis virus infection in the brain become increasingly enriched for viral antigen-specificity, suggesting initial recruitment of both specific and non-specific cells followed by preferential accumulation of virus-specific plasma cells over time (106, 112).

Within the parenchymal niche, B cells and plasma cells are found at microanatomical sites of active infection and inflammation, suggesting they are positioned to play a direct role in local protection and pathology. In an analysis of progressive multifocal leukoencephalopathy, subacute sclerosing panencephalitis, and cytomegalovirus encephalitis, IgG deposition was observed on virally infected cells, indicating targeting of virus-specific antibody and a potential role in clearance from the tissue (114). In MS, B cells and plasma cells accumulate in active parenchymal lesions as well as in perivascular spaces adjacent to these inflamed, demyelinating regions (30, 73, 74, 115–118). Recent work has even located plasma cells at the chronically inflamed “leading edge” of MS lesions (119). This localization, alongside IgG antibody deposition on myelin within lesions, alludes to a significant role for locally produced antibody in demyelination and disease pathology (114, 120). On the other hand, regulatory plasma cells have also been shown to be important for controlling autoimmune inflammation through IL-10 production in experimental autoimmune encephalomyelitis (EAE), the mouse model for MS (121). IgA-expressing plasma cells have even been shown to home into the CNS and produce immunoregulatory IL-10 to dampen neuroinflammation, raising the possibility that B lineage cells can be both pathogenic and protective in CNS autoimmune disease (4, 122).

Along with their presence in the parenchyma during infection and autoimmunity, plasma cells have also been identified in glial cell cancers, termed gliomas, in the CNS parenchyma (123, 124). These intratumoral plasma cells and their role in tumor immunity have largely gone unexplored. One study analyzing 25 different solid tumors, including glioblastoma, a common and aggressive glioma subtype, found intratumoral plasma cells to be correlated with improved survival (124). On the other hand, a more recent study focused on a glioblastoma patient cohort found that tumor-infiltrating plasma cells were associated with a poor prognosis (123). This study went on to show that IgG secreted by local plasma cells signaled to glioblastoma stem cells (GSCs) through IgG-FcɣRIIA-mTOR signaling which induced GSC proliferation. Blocking this axis reversed the phenotype, inhibiting GSC proliferation and self-renewal (123). Altogether, more work on intratumoral plasma cells in solid tumors within the CNS parenchyma will be necessary to understand the balance between their anti-tumor and pro-tumor regulatory roles.

Although often overlooked, the optic nerve and retina in the posterior region of the eye are critical CNS sites that have been studied in the context of autoimmune-driven optic neuritis and uveitis, respectively. Two common drivers of optic neuritis are MS and neuromyelitis optica (NMO). In a histological study of MS-driven disease, plasma cells were mainly found in the perivascular space within optic nerve lesions, with some cells also present in the parenchyma itself (125). In NMO-driven optic neuritis, we are unaware of work specifically detailing the plasma cell presence in the optic nerve; however, it is well established that AQP4-specific antibody, B cells, and plasma cells are present in NMO patient CSF (49, 60, 126). In addition, the antibody-mediated nature of astrocytopathy in NMO-driven optic neuritis is evidenced by antibody and complement deposition on astrocytic endfeet, where AQP4 is expressed, within parenchymal lesions (127, 128). This deposition was found to correspond with loss of AQP4 immunoreactivity and astrocytic impairment (127, 128). Lesional analysis across different CNS regions, including those in the optic nerve, suggests crucial plasma cell involvement in tissue pathology.

In the inflamed retina, plasma cell accumulation correlates with increased disease severity (129, 130). In experimental autoimmune uveitis (EAU), single cell analyses of ocular tissue plasma cells showed a heterogeneous mix of regulatory and pathogenic plasma cells that expressed many anti- and pro-inflammatory mediators, respectively (130). When plasma cells were systemically depleted using the proteasome inhibitor bortezomib, pathology was reduced, while transferring antigen-specific or the sorted pathogenic plasma cells directly into the eye exacerbated the disease severity (130, 131). While this study didn’t focus on the role of antibody production, plasma cells in the eye were seen to express both IgM and IgG (130). Altogether, there is strong evidence that plasma cells and humoral immunity are relevant drivers of ocular inflammation in the optic nerve and retina.

In persistent viral infection and chronic autoimmune inflammation, antigen-specific and non-specific plasma cell populations are maintained in the CNS suggesting either continuous replenishment of the antibody secreting cell pool from the periphery or formation of a local niche that supports prolonged plasma cell retention (30, 111). Class-switched plasma cells have been identified in the CNS of mice up to a year after viral infection (105, 106, 110, 111). Importantly, studies using different models of persistent viral brain infection have shown B lineage cells are crucial to controlling pathogen spread during the chronic phase of infection, underscoring their functional significance in sustaining long-term immune defense (107, 132).

In MS, plasma cells appear to be crucial in driving CNS pathology, as a lack of lesional plasma cells is associated with less severe disease (133). Despite this, Atacicept (TACI-Ig), a therapeutic that blocks BAFF (B cell activating factor) and APRIL (a proliferation-inducing ligand) signaling and drives the depletion of B lineage subsets, worsens MS disease and optic neuritis, and increases relapse rate (134, 135). While Atacicept, which effectively depletes both B cells and plasma cells by blocking shared survival signals, seems to exacerbate disease, CD20-based B cell depletion strategies that spare plasma cells have shown great efficacy against CNS autoimmunity. The unexpected Atacicept-mediated exacerbation of MS and optic neuritis may be partially explained by depleting regulatory CNS plasma cells (4). Subsequent studies showed that infiltrating monocytes produce APRIL, which activates astrocyte-intrinsic APRIL signaling and modulates CNS inflammation (136). In BCMA-deficient mice, loss of survival factor signaling contributes to the expansion of CNS-specific B cells (137). Each of these findings offer plausible explanations for why Atacicept exacerbates CNS autoimmunity. Altogether, the confounding, and sometimes seemingly contradictory, findings when targeting B lineage cells to treat CNS autoimmunity implicate their complex roles in driving and regulating autoimmune disease. Additional work will be necessary to delineate the mechanisms of these protective and pathogenic roles for future targeted therapies to exploit, or prevent, depending on the disease context.

## 4 Direct entry or local differentiation of plasma cells in the CNS and its borders?

One critical outstanding question in plasma cell biology is how they come to reside within the CNS and its borders. It is typically presumed that plasma cells differentiate in induction sites, generally secondary lymphoid organs (draining lymph nodes or spleen), then migrate via the blood into effector sites where they can locally produce antibody. However, data, particularly from the CNS, suggests this paradigm may be oversimplified. While plasma cells can express numerous chemokine receptors that theoretically facilitate tissue specific homing, so do B cells, and it is quite possible that CNS plasma cells are derived both from peripheral plasma cells emigrating into tissues via the blood as well as local B cell differentiation within effector tissues, particularly during CNS inflammation. Indeed, the original observations of oligoclonal plasma cells within the CNS is suggestive that these cells were derived locally from a limited (oligoclonal) number of B cell clones. With that said, most of the work we discuss here has not directly tested the relative roles of selective migration versus local differentiation, with experiments only suggestive of one hypothesis over the other.

In homeostasis, meningeal plasma cells express the chemokine receptor CXCR4 – a chemokine receptor known to be critical for plasma cell BM residence – while dural stromal cells express the CXCR4 ligand, CXCL12, providing a possible mechanism for local retention, much like in the BM (25, 33, 138). CXCL13 expression has also been detected in the meninges, CSF, and within inflamed MS lesions with a possible role in B lineage cell recruitment via CXCR5 signaling (40, 50, 139). Furthermore, age-associated meningeal accumulation of T-bet and CXCR3 expressing B cells suggests a possible role for CXCR3-driven migration into the tissue (33). These T-bet expressing cells clonally overlap with IgG+ plasma cells that accumulate alongside them (33). Together with the known propensity of T-bet expressing B cells to rapidly differentiate into plasma cells upon re-activation, this observation is suggestive, but not definitive, of local B cell differentiation into IgG+ plasma cells in the meninges (38, 39).

CXCR3 ligands CXCL9 and CXCL10 are commonly upregulated in the inflamed CNS (105). CXCL9 and CXCL10 production has been described for astrocytes and microglia within the CNS parenchyma, and soluble chemokines are detected in the CSF during both autoimmune disease and infection (49, 109). Expression of these CXCR3 ligands may be crucial both for parenchymal entry and localization to inflamed regions within the tissue. Both B cells and plasma cells that home into the CNS during inflammation express CXCR3 (49, 104, 105, 109). Accordingly, studies in JHMV infection show that both CXCL10 and CXCR3 deficiency led to diminished antibody secreting cell populations in the CNS (109, 140). Notably, loss of either CXCL9 or CXCL10 did not impact serum neutralizing antibody titers, implying CXCR3 signaling plays a role specifically in the CNS response (109). Thus, there is direct evidence suggesting CXCR3 is important for plasma cell accumulation in the CNS during viral infection.

Natalizumab, a therapeutic antibody that antagonizes the adhesive interaction between VLA4 and its ligands VCAM-1 and MADCAM-1, disrupts lymphocyte trafficking into the CNS and has shown dramatic efficacy in MS treatment (141). Intrathecal IgG is reduced in patients treated with Natalizumab, particularly in those who respond to treatment, suggesting a reduction of local plasma cells (142–144). At the same time, work has found persistence of oligoclonal bands in some patients, implying variability in the drug’s impact on local plasma cell subsets (143). In addition, reductions in CSF plasma cell frequency have been identified, with some studies attempting to emphasize the specific loss of plasmablasts (144–146). However, these studies rarely analyze longer-lived plasma cells themselves, limiting any interpretation about selective reduction of individual antibody secreting cell subsets. Indeed, plasma cells are also reported to be increased within parenchymal lesions after Natalizumab, suggesting that certain CNS antibody secreting cell subsets are not impacted by treatment (147). A definitive study on how Natalizumab impacts all subsets, from plasmablasts to long-lived plasma cells, may help explain variability in intrathecal antibody reduction and patient responses to the drug, as those with a higher frequency of long-lived plasma cells may be less responsive (144, 148). Furthermore, work has also found a reduction in CSF B cells, presenting the possibility that the impact of Natalizumab on intrathecal antibody may be in part due to a block of local B cell differentiation into CNS antibody secreting cells (144). Overall, these combined works suggest a potential role for VLA4 in the general accumulation of CNS antibody secreting cells while also indicating that long-lived plasma cells can be maintained within the CNS without continued recruitment from the periphery. ALCAM, or activated leukocyte cell adhesion molecule, has also been shown to facilitate B cell migration into the CNS (149).

Several studies provide circumstantial evidence for local differentiation by identifying proliferative (Ki67+) and clonally expanded B lineage cells across different CNS compartments (47, 51, 62, 106, 150). Work on Sindbis virus infection shows expansion of IgA+ plasma cells specifically within the CNS with no corresponding expansion in peripheral lymphoid tissue (106). In MS patients, an increase in CSF class-switched memory B cells was associated with a decrease in circulating class-switched memory B cells as well as a corresponding expansion of CSF antibody secreting cells (50). This suggested memory B cell migration from the blood into the CNS. When paired with the findings that antibody secreting cells responded poorly to migratory stimuli and were often sparse in circulation, this lends support for local differentiation of plasma cells in the CNS (50).

Moreover, the presence of class-switched memory B cells within autoimmune CNS lesions and the virally infected CNS parenchyma suggests that B cells with intrinsic differentiation potential are poised to locally generate plasma cells upon their reactivation (104). Understanding whether plasma cells are recruited into the CNS directly, or antigen-specific B cells are recruited via the blood and subsequently differentiate into plasma cells with the CNS, will require future experiments to directly test these respective possibilities. Because the blood brain barrier typically excludes circulating antibody from entering the CNS, understanding how antibody is “delivered” into the CNS will help guide future therapeutic interventions to prevent (or induce in the case of treating CNS tumors) plasma cell accumulation during autoimmune disease. In this regard, if B cells differentiate locally in the CNS, it may be necessary to specifically target B cell migration and entry into the tissue alongside plasma cell migration. Understanding if, and how, B cells and plasma cells differentially home into the tissue will also be important in this regard. In addition, treatments targeted towards depleting B cells and plasma cells within the CNS rather than in the periphery, using drugs like Rituximab and Atacicept, may even further improve efficacy in reducing CNS pathology.

## 5 Longevity of plasma cells in and around the CNS

The retention and longevity of plasma cells in the CNS and its border is needed to better understand the durability of protective and pathogenic humoral responses in the tissue. Pulse-chase experiments to label and track plasma cell survival over extended periods of time in these different tissues will be critical to improving our understanding in this area (151). Using EdU labelling to track cell survival over the “chase” period during active EAE found EdU-labelled plasma cells in the CNS parenchyma 5 weeks after administration. This provides direct evidence for long-lived plasma cell survival in the CNS (30). Further work using similar strategies to track plasma cells over longer time frames in different scenarios will better define the capacity and anatomical location for long term retention in these tissues.

Despite the limited work directly assessing plasma cell longevity, a number of factors suggest the potential for long-term CNS retention. In the meninges, CSF, and parenchyma, many factors associated with the plasma cell niche and survival have been identified during inflammation, including CXCL12, VCAM-1, BAFF, and APRIL (29, 30, 105). Specifically, human astrocytes have been shown both to upregulate BAFF *in vivo* in MS lesions and to secrete this key plasma cell survival factor after ex vivo treatment with IFN-g and TNF-a (152). This suggests astrocytes could be important regulators of plasma cell persistence during neuroinflammation.

Interestingly, BCMA (B cell maturation antigen), a receptor for both BAFF and APRIL expressed on the surface of plasma cells, is shed as soluble BCMA (sBCMA) into the CSF of MS patients (153, 154). Further, sBCMA levels were found to correlate with intracerebral IgG suggesting its CSF presence could serve as a biomarker for B lineage presence and function in CNS autoimmune disease (153, 154). Beyond use as a biomarker, the role for BCMA in CNS plasma cell survival and longevity is largely unknown. Studies on how BCMA signaling impacts long-lived plasma cell survival in the bone marrow and secondary lymphoid organs has been conflicting, with some work indicating a pro-survival role and some finding BCMA dispensable for plasma cell longevity (155–158). A recent study even noted increased plasma cells in the bone marrow after a boost immunization, leading the authors to suggest that BCMA’s main function is to regulate the antibody response by acting as a decoy receptor to limit the availability of key plasma cell survival factors (157). sBCMA prevalence in the CSF during MS suggests that this decoy mechanism could play a role in dampening the CNS plasma cell response. Supporting this, BCMA deficiency was found to exacerbate EAE in mice, with increased plasma cells in the CNS compartment (137). However, this work also saw broad alterations within other B lineage subsets in the absence of BCMA, most notably increases in regulatory B cells and IL-10 production, suggesting the impacts of BCMA are far-reaching and complex. Further work will be critical to better understand whether BCMA plays a direct role in plasma cell survival within and around the CNS.

During viral infection and autoimmunity, plasma cells take on a long-lived phenotype (CD19^-^) and populations are frequently found to persist from weeks to months (29, 30, 55, 107, 110, 130). However, plasma cell populations could be maintained either through long term plasma cell survival or continuous differentiation of short-lived plasma cells. Both virus-specific and non-specific plasma cells persist in the CNS, with work in Sindbis virus infection suggesting enrichment of specific cells over time (106, 112). Preferred retention of the virus-specific population might suggest antigen-driven selection of these cells and, therefore, a role for B cells in maintaining the population as opposed to long term plasma cell survival. Work on the mechanism of CD20-based B cell depletion therapies has shown continued maintenance of circulating and CSF antibody (159). This suggests long-lived, CD20^-^ plasma cell populations can reside within the CNS tissue. Altogether, evidence indicates that the CNS can potentially serve as a long-term niche for plasma cells, especially in chronic inflammation and infection, but further work will be needed to bring clarity.

While research on olfactory plasma cell longevity in the nasal mucosa itself remains scant, analysis of nasal wash samples following intranasal immunization has found overall nasal mucosal antibody responses to be durable in mice and humans (90, 92). Thus, there is evidence suggesting antibody secretion is maintained in the URT, but how long these URT plasma cells live in the tissue and how this specifically applies to the olfactory mucosa is yet to be determined. Further work determining plasma cell longevity in each of these tissues will be crucial for understanding the duration of antibody-based protection from infection. At the same time, elucidating how antibody production persists in pathological CNS immune responses will aid in the design of therapeutics to disrupt these responses.

## 6 Conclusion and future directions

Plasma cells, and the humoral arm of the immune system as a whole, have a dramatic impact on immunity in and around the CNS. But, by definition, humoral immunity relies on fluid to deliver it into tissues and yet specialized vascular barriers limit serum antibody access into the healthy CNS. Therefore, plasma cells must enter these tissues to deploy their antibodies locally. Plasma cells in the meninges and olfactory mucosa protect the CNS from neuroinvasive pathogens while antibody secretion within the CNS parenchyma can control established infection. At the same time, B lineage cells throughout the CNS niche are known to be crucial in both driving, and suppressing, autoimmune pathology in MS and other CNS-targeting autoimmune diseases. The “push pull’ potential for pro- and anti-inflammatory plasma cell functions are also important in CNS solid tumor responses; yet, in this context, pro-inflammatory anti-tumor plasma cell function is protective while regulatory plasma cell function is pathogenic. In each of these cases, local antibody production is needed in these CNS tissues as serum antibody generally cannot cross the blood brain barrier. Although the relevance of humoral immunity in these contexts is well established, there is still much that needs to be done to elucidate key knowledge gaps in plasma cell-driven defense and pathology in the CNS.

One significant gap surrounds the relative importance of plasma cell migration into the CNS versus local differentiation of plasma cells within the tissue. This is particularly relevant during CNS inflammation when antibody secreting cells rapidly accumulate. Another closely related gap is whether the meninges, olfactory mucosa, and/or CNS parenchyma serve as long lived plasma cell niches. Importantly, these two gaps will most likely not be filled by a single, all-encompassing answer, as unique disease contexts likely drive different levels of migration, local differentiation, and longevity. A third large gap in knowledge exists on plasma cells and humoral immunity in the olfactory mucosa. Very little work has focused on this specific mucosal tissue that is crucial for defense of both the URT and the CNS. While work in our lab and others has identified plasma cells in the olfactory mucosa of mice and humans, little is known about their migration and heterogeneity. Their relevance in preventing URT viral infection and subsequent neuroinvasion in a mouse model suggests potential for a prominent protective role in human infection. Understanding more about these plasma cells, from how they are generated, to their durability in the tissue, will be significant for future vaccine strategies against URT-tropic pathogens.

A concerted effort to fill the existing knowledge gaps surrounding plasma cells at CNS borders and within the CNS parenchyma will serve to bolster the field’s overall understanding of plasma cell heterogeneity and function in non-lymphoid tissues. Furthermore, it will aid in the development therapeutics to enhance protection of the CNS from infection and to prevent harmful autoimmune pathology.
